# Post-endoscopic Retrograde Cholangiopancreatography Pancreatitis after Conservative Treatment for Symptomatic Bile Duct Stones

**DOI:** 10.31662/jmaj.2022-0165

**Published:** 2023-03-13

**Authors:** Hirokazu Saito, Yoshihiro Kadono, Takashi Shono, Kentaro Kamikawa, Atsushi Urata, Jiro Nasu, Masayoshi Uehara, Ikuo Matsushita, Tatsuyuki Kakuma, Shuji Tada

**Affiliations:** 1 Department of Gastroenterology, Kumamoto City Hospital, Kumamoto, Japan; 2 Department of Gastroenterology, Tsuruta Hospital, Kumamoto, Japan; 3 Department of Gastroenterology, Kumamoto Chuo Hospital, Kumamoto, Japan; 4 Department of Gastroenterology, Saiseikai Kumamoto Hospital, Kumamoto, Japan; 5 Department of Gastroenterological Surgery, Kumamoto Chuo Hospital, Kumamoto, Japan; 6 Department of Biostatics Center, Medical School, Kurume University, Kurume, Japan

**Keywords:** endoscopic retrograde cholangiopancreatography, bile duct stone, post-endoscopic retrograde cholangiopancreatography pancreatitis, asymptomatic patients

## Abstract

**Introduction:**

Endoscopic retrograde cholangiopancreatography (ERCP) for asymptomatic common bile duct stones (CBDS) has been associated with an increased risk of post-ERCP pancreatitis (PEP). Patients with asymptomatic CBDS at the time of ERCP include those with incidentally discovered CBDS (group A) and previously symptomatic patients with CBDS who became asymptomatic after conservative treatment for symptomatic CBDS, including obstructive jaundice or acute cholangitis (group B). In this study, we aimed to examine PEP risk in group B by comparing PEP risks between groups A, B, and currently symptomatic patients (group C).

**Methods:**

In this multicenter retrospective study, we examined 77 patients in group A, 41 patients in group B, and 1225 patients in group C who had native papillae. PEP incidence rates between asymptomatic patients at the time of ERCP (groups A and B) and symptomatic patients (group C) were compared using one-to-one propensity score matching. Bonferroni’s correction analysis was also performed to compare PEP incidence rates among the three groups.

**Results:**

As per our findings, PEP incidence rate in propensity score-matched groups A and B was significantly higher than that of propensity score-matched group C (13.2% [15/114] versus 4.4% [5/114], respectively, P = 0.033). In groups A and B, PEP incidence rates were 11.7% (9/77) and 14.6% (6/41), respectively. PEP risk in group B was similar to that in group A (P = 1.0). PEP incidence in group B was significantly higher than PEP incidence in group C (14.6% (6/41)) vs. 2.9% (35/1225)) (P = 0.005)).

**Conclusions:**

ERCP for previously symptomatic patients with CBDS who became asymptomatic after conservative treatment for symptomatic CBDS may increase the risk of PEP compared with ERCP for currently symptomatic patients. Thus, ERCP should be performed before patients become asymptomatic using conservative treatments if patients can tolerate ERCP procedures.

## Introduction

Recently, higher risk of post-endoscopic retrograde cholangiopancreatography (ERCP) pancreatitis (PEP) has been reported in patients with asymptomatic common bile duct stones (CBDS) as compared with patients with symptomatic CBDS ^(1), (2), (3), (4)^. As per previous reports, PEP incidence rates in asymptomatic and symptomatic patients with CBDS were 7.6%-20.8% and 3.0%-6.9%, respectively ^(1), (2), (3), (4), (5)^.

Although asymptomatic CBDS has been typically defined as CBDS without noticeable symptoms and hematologic abnormalities, and incidentally discovered using imaging modalities for other diseases, some patients can become asymptomatic at the time of ERCP after conservative treatment for symptomatic CBDS, including obstructive jaundice and acute cholangitis. We hypothesized that ERCP should be performed when patients are symptomatic if the risk of PEP for patients with CBDS who became asymptomatic after conservative treatment for symptomatic CBDS is comparable with that of patients with incidentally discovered CBDS. However, there are yet no studies examining PEP risks in patients who became asymptomatic after conservative treatment for symptomatic CBDS. Thus, in this study, we aim to examine the risks of PEP in patients who became asymptomatic at the time of ERCP after conservative treatment for symptomatic CBDS.

## Materials and Methods

### Study design

In this multicenter retrospective study, we reviewed the electronic medical records of 1343 patients who were treated with ERCP for CBDS from April 2012 to February 2020 at three institutions, namely, Kumamoto City Hospital, Kumamoto Chuo Hospital, and Saiseikai Kumamoto Hospital, Japan. We compared PEP incidences between patients with asymptomatic CBDS incidentally discovered using an imaging modality for other diseases (group A, n = 77); previously symptomatic patients with CBDS who became asymptomatic after conservative treatment for symptomatic CBDS, such as obstructive jaundice or acute cholangitis (group B, n = 41); and currently symptomatic patients with CBDS (group C, n = 1225). Patient data in this study were similar to the data used in our previous reports on PEP risks in asymptomatic patients and the disease-based risk stratification of PEP for CBDS ^(2), (6)^.

The inclusion criteria were as follows: (1) patients with native major duodenal papilla, (2) patients with normal gastrointestinal tract or Billroth-1 reconstruction, and (3) patients confirmed to have CBDS during ERCP. The exclusion criteria were as follows: (1) patients who had experienced ERCP, (2) patients with Billroth-II or Roux-en-Y reconstruction, (3) patients without CBDS during ERCP, and (4) patients with some symptoms identified to be CBDS via an imaging modality before cholecystectomy for gallstones because gallstone-related and CBDS-related symptoms, such as fever and upper abdominal pain, were similar; thus, it was difficult to determine whether patients’ symptoms originated from gallstones or CBDS.

Ethics Review Committees of Kumamoto City Hospital (approval number: 582), Kumamoto Chuo Hospital (approval number: 70-02), and Saiseikai Kumamoto Hospital (approval number: 855) provided approval for this study, and opt-out consent was used.

### Study definitions

#### Groups A, B, and C

Patients with asymptomatic CBDS incidentally discovered via an imaging modality for other diseases, who had no history of CBDS-related symptoms and hematologic abnormalities, were defined as group A. Exceptionally, patients with constitutional jaundice, who had asymptomatic CBDS, were included in group A if they had no history of CBDS-related symptoms and hematologic abnormalities, except for elevated total serum bilirubin levels. Previously symptomatic patients with a history of CBDS-related symptoms and/or hematologic abnormalities, who became asymptomatic after conservative treatment for symptomatic CBDS, such as obstructive jaundice or acute cholangitis, were included in group B. Currently symptomatic patients with acute cholangitis and elevated hepatobiliary enzymes without cholangitis were defined as symptomatic CBDS (group C). Acute cholangitis was diagnosed and graded based on the consensus criteria of the revised Tokyo Guidelines (2018) ^(7)^.

#### PEP

We used the consensus criteria of Cotton et al. to diagnose and grade PEP ^(8)^.

### ERCP procedures

All ERCP procedures were performed by 38 different endoscopists, including 18 non-expert endoscopists with an experience of <200 ERCP procedures, using a side-viewing duodenoscope (Olympus JF-260, TJF-260V; Olympus Medical Systems, Tokyo, Japan) in patients sedated with pethidine hydrochloride and midazolam. After CBDS were confirmed via a cholangiogram, endoscopic sphincterotomy, endoscopic papillary balloon dilation (EPBD), or endoscopic papillary large-balloon dilation procedures were performed to open the bile duct orifice. CBDS were removed using a basket catheter, a balloon catheter, and/or endoscopic mechanical lithotripsy. When biliary drainage was required, endoscopic retrograde biliary drainage or endoscopic nasobiliary drainage procedures were performed.

**Figure 1.**
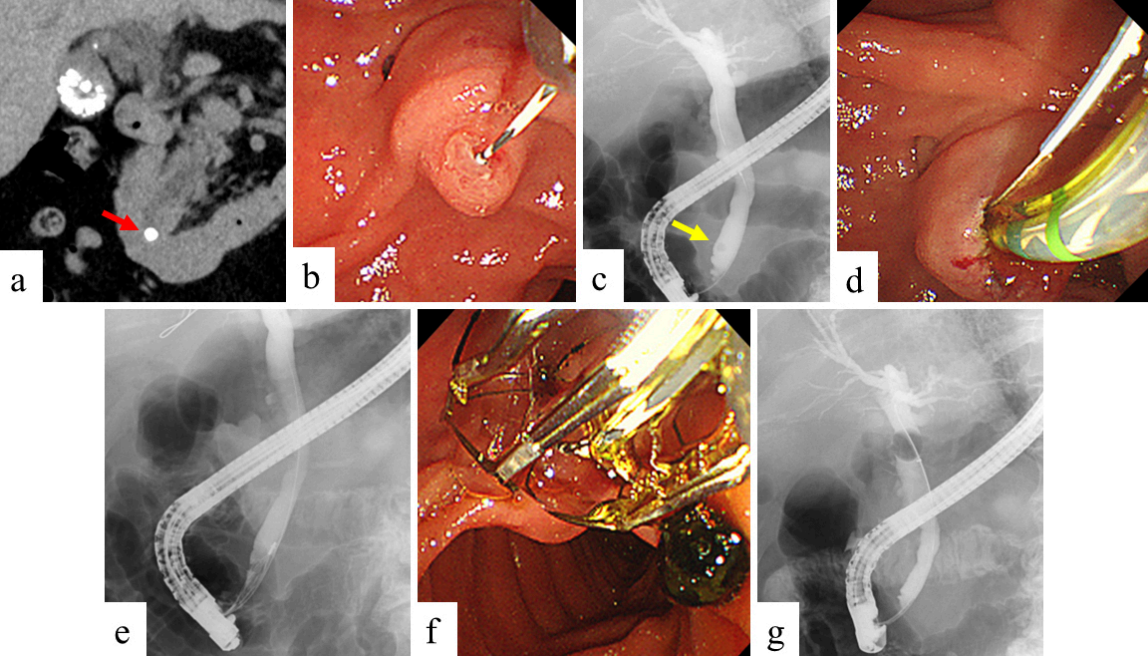
Preprocedural images and images during ERCP in group A. a. Computed tomography image. A common bile duct stone with a diameter of 5 mm was observed in distal bile duct. (red arrow). b. Guidewire was inserted into the common bile duct after selective biliary cannulation. c. Cholangiogram. A common bile duct stone with a diameter of 5 mm was observed in distal bile duct. (yellow arrow). d. Endoscopic sphincterotomy was performed to extract the stone. e, f. A common bile duct stone was completely extracted using a basket catheter. g. No residual stones were observed via cholangiogram using a balloon catheter.

**Figure 2.**
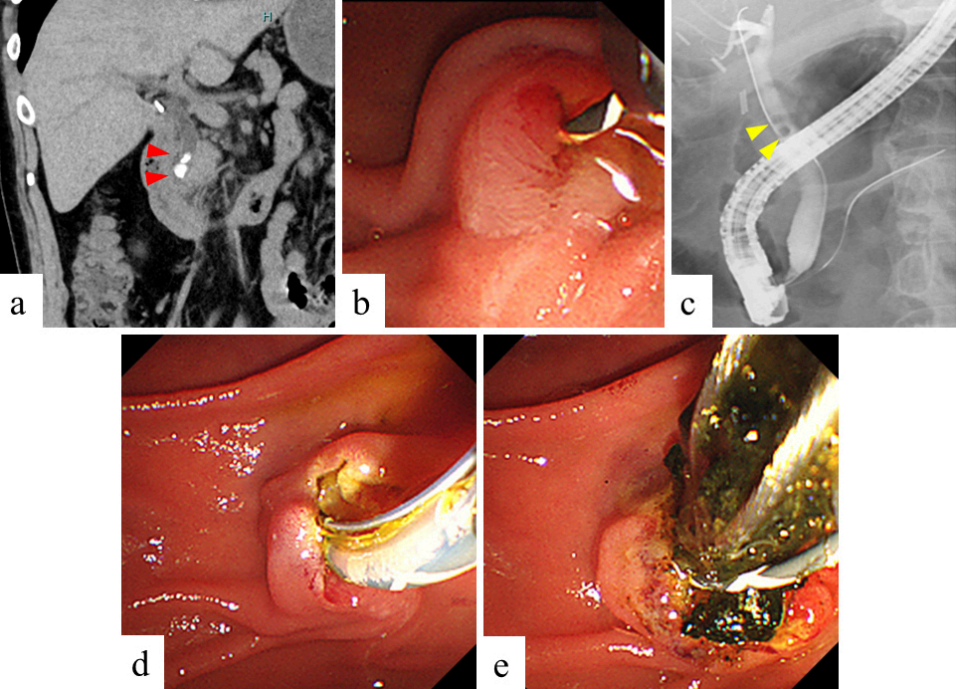
Preprocedural images and images during ERCP in group B. a. Computed tomography image. Two common bile duct stones with a diameter of 6 mm and 4 mm were observed in distal bile duct. (red arrowheads). b. Guidewire was inserted into the common bile duct after selective biliary cannulation. c. Cholangiogram. Two common bile duct stones with a diameter of 6 mm and 4 mm were observed in upper bile duct. (yellow arrowheads). d. Endoscopic sphincterotomy was performed to extract the stones. e. Common bile duct stones were completely extracted using a basket catheter.

A prophylactic pancreatic stent for PEP occurrence was inserted, based on operator decision. Non-expert endoscopists were supervised by an experienced endoscopist to ensure ERCP safety and reliability. We usually administer 1500-2000 mL of lactated Ringer’s solution during the fasting period of 12-24 h after ERCP in patients without contraindications for hydration. Preprocedural images and images during ERCP in groups A and B are shown in Figure 1 and 2, respectively.

#### Statistical analysis

Chi-squared or Fisher’s exact tests were used to compare categorical variables, whereas the Welch’s t-test was used to compare continuous variables. Bonferroni adjustments were used for multiple comparisons. A one-to-one propensity score matching analysis with a caliper of 0.2 was applied to adjust the patient characteristics between asymptomatic patients at the time of ERCP (groups A and B) and currently symptomatic patients (group C). The propensity scores were then calculated with a multivariate logistic regression model using all the confounders presented in Table 1. Statistical significance was accepted at two-sided p-values <0.05. All statistical analyses were performed using EZR software version 1.53 (Saitama Medical Center, Jichi Medical University, Saitama, Japan) and a graphical user interface for R software (The R Foundation for Statistical Computing, Vienna, Austria, version 4.1.2) ^(9)^.

**Table 1.** Patient Characteristics of Asymptomatic Patients at the Time of ERCP (Groups A and B) and Symptomatic Patients (Group C).

	All patients					Propensity score-matched groups				
	Groups A and B		Group C		P-value	Groups A and B		Group C		P-value
	(n = 118)		(n = 1225)			(n = 114)		(n = 114)		
Age [mean (SD)]	74.8	(9.8)	75.0	(14.4)	0.85	75.0	(9.7)	74.4	(15.0)	0.70
Female (%)	55	(46.6)	581	(47.4)	0.92	54	(47.4)	48	(42.1)	0.51
Dialysis (%)	4	(3.4)	36	(2.9)	0.77	4	(3.5)	3	(2.6)	1.0
Billroth-1 reconstruction (%)	6	(5.1)	30	(2.4)	0.12	6	(5.3)	6	(5.3)	1.0
Non-expert endoscopists (%)	24	(20.3)	184	(15.0)	0.14	22	(19.3)	15	(13.2)	0.28
Successful biliary cannulation (%)	115	(97.5)	1212	(98.9)	0.16	111	(97.4)	112	(98.2)	1.0
Periampullary diverticulum (%)	38	(32.2)	367	(30.0)	0.60	37	(32.5)	30	(26.3)	0.38
Normal serum bilirubin (%)	112	(94.9)	461	(37.6)	<0.001	108	(94.7)	109	(95.6)	1.0
Non-dilated CBD (<10 mm) (%)	41	(34.7)	494	(40.3)	0.28	40	(35.1)	39	(34.2)	1.0
Contrast-assisted cannulation (%)	87	(73.7)	876	(71.5)	0.67	84	(73.7)	82	(71.9)	0.88
Wire-guided cannulation (%)	8	(6.8)	126	(10.3)	0.26	8	(7.0)	7	(6.1)	1.0
PGW-assisted cannulation (%)	11	(9.3)	166	(13.6)	0.25	10	(8.8)	11	(9.6)	1.0
Difficult biliary cannulation >10min (%)	43	(36.4)	289	(23.6)	0.003	40	(35.1)	39	(34.2)	1.0
Pancreatic injection (%)	60	(50.8)	533	(43.5)	0.15	57	(50.0)	58	(50.9)	1.0
Precut sphincterotomy (%)	12	(10.2)	56	(4.6)	0.014	12	(10.5)	14	(12.3)	0.84
Use of balloon catheter (%)	94	(79.7)	979	(79.9)	0.91	92	(80.7)	90	(78.9)	0.87
Use of basket catheter (%)	65	(55.1)	553	(45.1)	0.042	62	(54.4)	61	(53.5)	1.0
Mechanical lithotripsy (%)	17	(14.4)	197	(16.1)	0.70	16	(14.0)	16	(14.0)	1.0
Biliary stent placement (%)	83	(70.3)	1072	(87.5)	<0.001	82	(71.9)	85	(74.6)	0.77
EST (%)	86	(72.9)	893	(72.9)	1.0	82	(71.9)	80	(70.2)	0.88
EPBD (%)	7	(5.9)	132	(10.8)	0.11	7	(6.1)	8	(7.0)	1.0
EPLBD (%)	22	(18.6)	187	(15.3)	0.35	22	(19.3)	24	(21.1)	0.87
Prophylactic pancreatic stent placement (%)	16	(13.6)	166	(13.6)	1.0	16	(14.0)	15	(13.2)	1.0
Rectal NSAIDs (%)	10	(8.5)	108	(8.8)	1.0	9	(7.9)	8	(7.0)	1.0
Post-cholecystectomy (%)	27	(22.9)	129	(10.5)	<0.001	25	(21.9)	30	(26.3)	0.54
Presence of gallstones (%)	60	(50.8)	759	(62.0)	0.023	58	(50.9)	56	(49.1)	0.90
Large stones (>10 mm) (%)	14	(11.9)	234	(19.1)	0.062	14	(12.3)	15	(13.2)	1.0
Number of CBDS [mean (SD)]	2.7	(3.9)	2.2	(2.5)	0.15	2.8	(4.0)	2.5	(3.0)	0.54
Procedure time, min [mean (SD)]	31.5	(18.8)	26.1	(14.9)	0.003	31.5	(19.0)	31.2	(10.8)	0.91

CBD, common bile duct; PGW, pancreatic guidewire; EST, endoscopic sphincterotomy; EPBD, endoscopic papillary balloon dilation; EPLBD, endoscopic papillary large balloon dilation; NSAIDs, nonsteroidal anti-inflammatory drugs; CBDS, common bile duct stones

## Results

### Diagnostic modalities for CBDS before ERCP

In total, 183 patients were diagnosed with CBDS before ERCP by magnetic resonance cholangiopancreatography (MRCP), 858 by computed tomography (CT), 64 by abdominal ultrasonography (US), 12 by endoscopic ultrasonography, and 226 by more than two modalities.

### Characteristics of asymptomatic patients at the time of ERCP (groups A and B) and symptomatic patients (group C)

The characteristics of the asymptomatic patients at the time of ERCP (groups A and B) and symptomatic patients (group C) are presented in Table 1. In all patients, normal serum bilirubin levels, difficult biliary cannulation >10 min, precut sphincterotomy, use of basket catheter, post-cholecystectomy, and prolonged procedure time were more frequently observed in groups A and B than in group C. Meanwhile, biliary stent placement and the presence of gallstones were observed more frequently in group C than in groups A and B. In the propensity score-matched group, no significant differences were observed between groups A and B and group C.

### PEP incidence rates in asymptomatic patients at the time of ERCP (groups A and B) and symptomatic patients (group C)

PEP incidence rates among asymptomatic patients at the time of ERCP (groups A and B) and symptomatic patients (group C) are presented in Table 2. In all patients, the PEP incidence rates in groups A and B and in group C were 12.7% (15/118) and 2.9% (35/1225), respectively. The PEP incidence rate in groups A and B was noted to be significantly higher than that in group C (P < 0.001). In the propensity score-matched group, the PEP incidence rates in groups A and B and in group C were 13.2% (15/114) and 4.4% (5/114), respectively. The PEP incidence rate in the propensity score-matched groups A and B was significantly higher than that in the propensity score-matched group C (P = 0.033).

**Table 2.** PEP Incidence Rates in Asymptomatic Patients at the Time of ERCP (Groups A and B) and Symptomatic Patients (Group C).

	All patients			Propensity score-matched groups		
	Groups A and B	Group C	P-value	Groups A and B	Group C	P-value
	(n = 118)	(n = 1225)		(n = 114)	(n = 114)	
PEP (%)	15 (12.7)	35 (2.9)	<0.001	15 (13.2)	5 (4.4)	0.033

PEP, post-endoscopic retrograde cholangiopancreatography pancreatitis

### Patient characteristics of groups A and B

The patient baseline characteristics of groups A and B are presented in Table 3. No differences in terms of patient baseline characteristics were noted in groups A and B. All patients in group B were referred to our hospital after receiving conservative therapy for acute cholangitis or obstructive jaundice at other clinics. Moreover, 4 of the 41 patients in group B had a history of mild acute cholangitis before being referred to our hospital. Most patients in group B were suspected to have CBDS but were not definitively diagnosed with CBDS at clinics before referral to our hospital.

**Table 3.** Patient Characteristics in Groups A and B.

	Group A	Group B	P-value
	(n = 77)	(n = 41)	
Age [mean (SD)]	74.3 (9.1)	75.8 (11.0)	0.47
Female (%)	33 (42.9)	22 (53.7)	0.33
Dialysis (%)	3 (3.9)	1 (2.4)	1.0
Billroth-1 reconstruction (%)	3 (3.9)	3 (7.3)	0.42
Non-expert endoscopists (%)	17 (22.1)	7 (17.1)	0.63
Successful biliary cannulation (%)	74 (96.1)	41 (100.0)	0.55
Periampullary diverticulum (%)	25 (32.5)	13 (31.7)	1.0
Normal serum bilirubin (%)	74 (96.1)	38 (92.7)	0.42
Non-dilated CBD (<10 mm) (%)	28 (36.4)	13 (31.7)	0.69
Contrast-assisted cannulation (%)	56 (72.7)	31 (75.6)	0.83
Wire-guided cannulation (%)	7 (9.1)	1 (2.4)	0.26
PGW-assisted cannulation (%)	7 (9.1)	4 (9.8)	1.0
Difficult biliary cannulation >10min (%)	29 (37.7)	14 (34.1)	0.84
Pancreatic injection (%)	42 (54.5)	18 (43.9)	0.34
Precut sphincterotomy (%)	7 (9.1)	5 (12.2)	0.75
Use of balloon catheter (%)	60 (77.9)	34 (82.9)	0.63
Use of basket catheter (%)	43 (55.8)	22 (53.7)	0.85
Mechanical lithotripsy (%)	9 (11.7)	8 (19.5)	0.28
Biliary stent placement (%)	56 (72.7)	27 (65.9)	0.53
EST (%)	59 (76.6)	27 (65.9)	0.28
EPBD (%)	2 (2.6)	5 (12.2)	0.049
EPLBD (%)	13 (16.9)	9 (22.0)	0.62
Prophylactic pancreatic stent placement (%)	12 (15.6)	4 (9.8)	0.57
Rectal NSAIDs (%)	7 (9.1)	3 (7.3)	1.0
Post-cholecystectomy (%)	16 (20.8)	11 (26.8)	0.50
Presence of gallstones (%)	41 (53.2)	19 (46.3)	0.56
Large stones (> 10 mm) (%)	10 (13.0)	4 (9.8)	0.77
Number of CBDS [mean (SD)]	2.4 (3.0)	3.3 (5.2)	0.33
Procedure time, min [mean (SD)]	32.3 (18.8)	30.0 (19.0)	0.53

CBD, common bile duct; PGW, pancreatic guidewire; EST, endoscopic sphincterotomy; EPBD, endoscopic papillary balloon dilation; EPLBD, endoscopic papillary large balloon dilation; NSAIDs, nonsteroidal anti-inflammatory drugs; CBDS, common bile duct stones

### PEP incidence rates and severity values in groups A and B

PEP incidence rates and severity values in groups A and B are shown in Table 4. In group A and group B, PEP incidence rates were 11.7% (9/77) and 14.6% (6/41), respectively. We observed no significant differences between group A and group B (P = 0.77). Furthermore, we noted no significant differences in terms of PEP severity between group A and group B (P = 1.0).

**Table 4.** Comparing the Incidence and Severity of Post-ERCP Pancreatitis between Groups A and B.

	Group A	Group B	P-value
	(n = 77)	(n = 41)	
Post-ERCP pancreatitis (%)	9 (11.7%)	6 (14.6%)	0.77
Severity			1.0
Mild (%)	5 (55.6%)	3 (50.0%)	
Moderate (%)	3 (33.3%)	3 (50.0%)	
Severe (%)	1 (11.1%)	0 (0%)	

ERCP, endoscopic retrograde cholangiopancreatography

### Multiple comparisons of PEP incidences among groups A, B, and C

**Figure 3.**
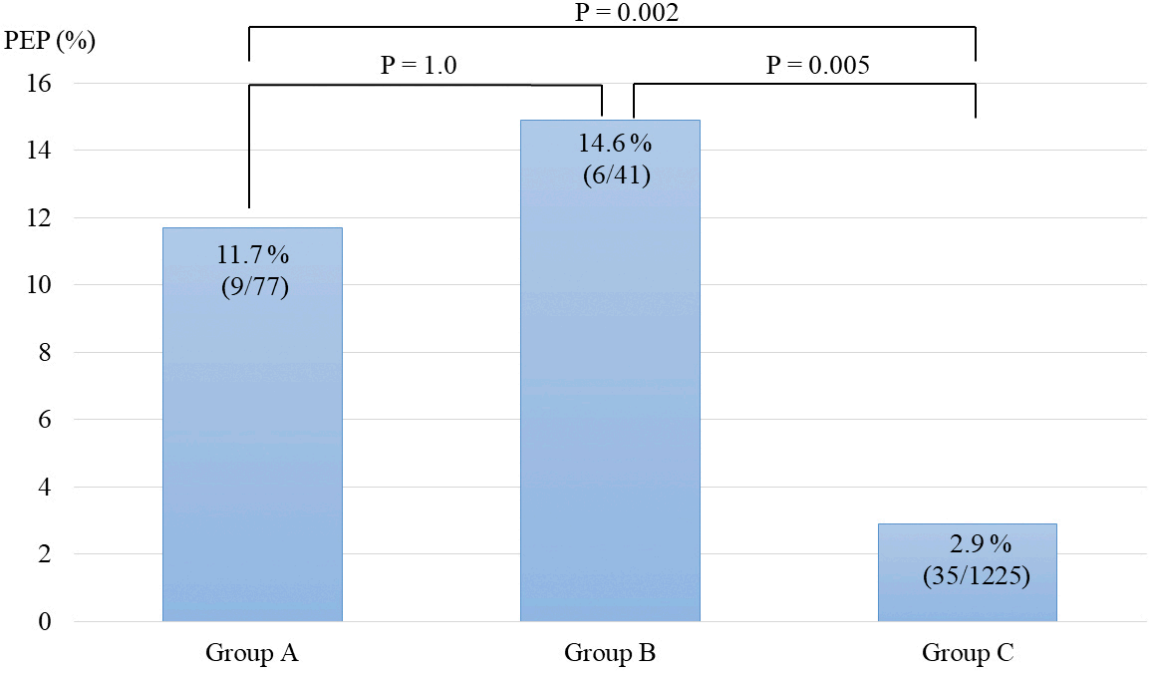
Multiple comparisons of the post-endoscopic retrograde cholangiopancreatography pancreatitis incidence among groups A, B, and C.

Comparisons of PEP incidence rates among groups A, B, and C are shown in Figure 3. The PEP incidence in group A and group B exhibited no significant differences (P = 1.0). The PEP risk in group B was found to be significantly higher than that in group C (P = 0.005).

## Discussion

Endoscopic treatment using ERCP has been strongly indicated for symptomatic CBDS, such as acute cholangitis and cholestasis without cholangitis. However, conservative treatment may be opted for some patients with symptomatic CBDS, and ERCP may be performed after improving patient symptoms and abnormal hematologic tests in institutions or clinics where ERCP cannot be performed immediately, or in elderly patients and patients with a poor performance status.

Recently, it was reported that the PEP incidence risk was higher in patients with asymptomatic CBDS than in patients with symptomatic CBDS, as well-known patient- and procedure-related PEP risk factors, such as normal serum bilirubin, non-dilated bile duct, and difficult biliary cannulation, were more common in patients with asymptomatic CBDS than those with symptomatic CBDS ^(1), (2), (3), (4)^. In our previous report, PEP was found to occur in 3.0% (28/949) of symptomatic patients and in 14.6% (24/164) of asymptomatic patients ^(2)^. Other studies have reported that PEP incidence rates in asymptomatic and symptomatic patients with CBDS were 7.6%-20.8% and 3.0%-6.9%, respectively ^(1), (2), (3), (4), (5)^. Furthermore, we reported that the PEP risk can vary with respect to the CBD-related disease; the PEP risk in patients with CBDS increased according to acute cholangitis, obstructive jaundice without acute cholangitis, and asymptomatic CBDS ^(6)^. Therefore, we hypothesized that the PEP risk for patients who became asymptomatic after conservative treatment for symptomatic CBDS may increase as compared with the PEP risk for symptomatic patients.

In this study, we have showed that patient characteristics associated with PEP risk, such as normal serum bilirubin and non-dilated CBD, were similar in group A and group B, as conservative treatment improved cholestasis in symptomatic patients. Furthermore, the well-known procedure-related PEP risk factor, that is, difficult biliary cannulation, was both noted in group A and group B. As a result, the PEP incidence in group B was similar to group A. Furthermore, considering the PEP risk was significantly higher in group B than in group C, ERCP should be performed when CBDS patients are symptomatic.

As ERCP is noted to be a challenging procedure, it can only be performed in specialized centers; therefore, conservative treatment may be selected for symptomatic CBDS in institutions wherein ERCP cannot be performed immediately. However, if patients can tolerate ERCP procedures, those with symptomatic CBDS and had received conservative therapy should be referred to a specialized center before they become asymptomatic. Furthermore, although conservative treatment may be selected for acute cholangitis or obstructive jaundice without definitive diagnosis of the cause by imaging in clinics, the clinicians, without hesitation, should refer the patients to specialized centers in order to diagnose the cause of acute cholangitis and obstructive jaundice and determine the indication of ERCP.

In elderly patients and patients with a poor performance status, conservative therapy may be used. It was previously reported that the incidence of procedure-related complications and efficacy of ERCP in elderly patients aged over 80-90 years old with poor performance status were comparable with those of patients under 80 years old with good performance status scores ^(10), (11), (12), (13), (14), (15)^. However, ERCP-related complication severity was reported as more severe in elderly patients with poor performance status ^(16), (17)^. If patients can tolerate endoscopic treatment by ERCP based on patient condition, ERCP should be performed when patients were symptomatic considering the PEP risk. Furthermore, we have previously reported that ERCP for patients without acute cholangitis had a higher PEP risk than patients with acute cholangitiz ^(18)^. In particular, early ERCP intervention should be considered for patients with acute cholangitis before any procedure to improve acute cholangitis to reduce the risk of PEP.

While it is deemed ideal to perform ERCP when patients are symptomatic, there may be opportunities to perform ERCP after symptomatic patient with CBDS became asymptomatic by conservative treatment. A retrospective study suggested that PEP risk factors in patients with asymptomatic CBDS involved trainees, EPBD, and precut sphincterotomy ^(19)^. Another study has reported that ERCP safety for asymptomatic CBDS by experienced endoscopists was comparable with ERCP safety for symptomatic CBDS ^(5)^. Therefore, ERCP for patients who became asymptomatic after conservative treatment for symptomatic CBDS should be performed by experienced endoscopists. In high-risk PEP patients, such as those who underwent precut sphincterotomy and EPBD, prophylactic pancreatic stent placement and aggressive hydration should be considered ^(20)^.

This study has limitations, and this includes the retrospective nature of the study and the small number of patients in group B.

In conclusion, ERCP for patients who became asymptomatic after conservative treatment for symptomatic CBDS may increase the risk for PEP compared with that in patients who are symptomatic. Although conservative therapy may be used in some instances, ERCP should be performed when patients with CBDS are symptomatic, but only if patients can tolerate ERCP procedures.

## Article Information

### Conflicts of Interest

None

### Acknowledgement

We would like to thank the staff involved in ERCP at the participating institutions.

### Author Contributions

All authors contributed to the conception and design of this study. Hirokazu Saito, Yoshihiro Kadono, and Tatsuyuki Kakuma collected and interpreted the data. Hirokazu Saito wrote the original draft. Takashi Shono, Kentaro Kamikawa, Atsushi Urata, Jiro Nasu, Masayoshi Uehara, and Ikuo Matsushita made an important contribution for writing-review and editing. Shuji Tada supervised the entire process.

### Approval by Institutional Review Board (IRB)

Ethics Review Committees of Kumamoto City Hospital (approval number: 582), Kumamoto Chuo Hospital (approval number: 70-02), and Saiseikai Kumamoto Hospital (approval number: 855).
